# Cerebellar infarction after head injury

**DOI:** 10.4103/0974-2700.62102

**Published:** 2010

**Authors:** Amit Agrawal, Anand Kakani

**Affiliations:** Department of Neurosurgery, Datta Meghe Institute of Medical Sciences, Sawangi (Meghe), Wardha, India

Sir,

In patients of stroke, the incidence of cerebellar infarction is approximately 1.5%.[1] The common causes of cerebellar infarction include arterial occlusion as a result of intracranial vertebral artery dissection (40%), cardioembolism (due to patent foramen ovale, rheumatic valvular disease, etc.), and, less commonly, hematologic disturbances and migraine, penetrating injuries of the vertebral artery,[2] and forceful abrupt cervical hyperextension (a common chiropractic maneuver).[3] Posttraumatic cerebellar infarction following head injury is uncommon, with only a few case reports in the literature.[4–6]

**Figure 1:**
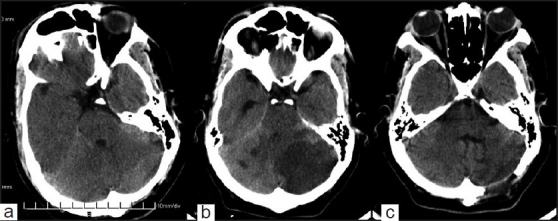
(a) CT scan showing right cerebellar infarction, (b) follow-up CT scan showing infarction, and (c) postoperative scan showing the opened up ventricle

A 46-year-old gentleman met with an accident while driving a motorcycle. At the time of the accident, he had no head support and was not wearing a helmet. Following the fall, he had had transient loss of consciousness and 2-3 episodes of vomiting. There was no history of seizures or of bleeding from the ear, nose, or mouth. His general and systemic examination was normal. He was drowsy but rousable. The Glasgow Coma Score (GCS) was E3V5M6. The pupils were bilaterally equal and reacting to light. Cranial nerves examination was normal. There were no focal motor neurological deficits. The initial CT scan (performed 6 h after injury) was normal [Figure 1a]. X-ray cervical spine (anteroposterior and lateral view) was normal.

The patient was managed conservatively. the next day his sensorium improved. He, however, complained of headache. Forty-eight hours after the injury, the patient became progressively drowsy and had multiple episodes of vomiting. The GCS was E2V4M5. The pupils were equal and reacting to light. The full range of ocular movements was present and he was moving all four limbs. Follow-up CT scan showed a large low-density area occupying the most of the left cerebellum. The fourth ventricle was shifted to the right, and the third and fourth ventricles were dilated. These features were suggestive of cerebellar infarction and obstructive hydrocephalus [Figure 1b]. A CT angiogram of the neck vessels could not be performed because the facilities were not available and the patient could not afford to do the investigation elsewhere. In view of the rapid neurological deterioration and the presence of a large cerebellar infarct, the patient underwent an emergency left paramedian suboccipital craniectomy and decompression of the infarcted cerebellum. He made an uneventful recovery. Follow-up CT scan showed opening up of the fourth ventricle and reduction in the size of the ventricles [Figure 1c]. Follow-up color Doppler study of the neck vessels, electrocardiogram, and echocardiography were all normal.

The mechanisms whereby blood supply to the cerebellar tissue is compromised following trauma and resultant infarction include trauma to the vertebral artery, with tearing of the intima and thrombus formation (dissection of the vertebral artery)[3,6,7] and embolism or hemodynamic failure.[8] A rare case has been described in which severe local trauma with an instantaneous deformity of bone resulted in an injury to the cerebellar cortical artery, leading to the cerebellar infarction under the occipital bone.[4] We hypothesize that a similar mechanism resulted in the cerebellar infarction in the present case. The early clinical features of cerebellar infarction are similar to that of any other intrinsic cerebellar lesion: the symptoms include headache, dizziness, nausea, vomiting, and loss of balance, and the signs are truncal and appendicular ataxia, nystagmus, and dysarthria.[9–11] However, as the cerebellar edema progresses the patient manifests the clinical features of compression of the brain stem and obstructive hydrocephalus.[9–11] Initially, the Babinski sign becomes positive and there is hemiparesis, profound drowsiness, and small but still reactive pupils; this is followed within hours by coma, posturing, or flaccidity and ataxic respirations.[10–12] Although MRI provides early and accurate visualization of cerebellar infarction, CT scan is more useful in patients with head injury. On plain CT scan cerebellar infarction appears as an area of focal hypodensity in the cerebellum, with or without compression of the fourth ventricle.[13] When available, multidetector CT angiography is a noninvasive, highly specific, and sensitive imaging modality to rule out vertebral artery dissection. It can be used as a supplement to unenhanced brain CT to rule out vertebral artery dissection.[14] If not provided with surgical intervention, about 80% of patients who have developed signs of brain stem compression will die, usually within hours to a few days.[9,12] Anti-edema measures (steroids, mannitol, and hyperventilation) may not be effective in reducing the edema that follows cerebellar infarction.[15] There is considerable literature to support suboccipital decompression and removal of the posterior rim of the foramen magnum to relieve tonsillar herniation, with removal by suction of grossly infarcted cerebellar tissue) as soon as signs of direct brain stem compression appear.[5,9,10,12,16] Where there is associated hydrocephalus, ventricular drainage alone – without prompt suboccipital decompression – will not relieve the direct compression of the brain stem; on the contrary, there will be a risk of upward herniation with ventriculostomy.[9]
